# Effect of introducing interprofessional education concepts on students of various healthcare disciplines: a pre-post study in the United Arab Emirates

**DOI:** 10.1186/s12909-022-03571-9

**Published:** 2022-07-02

**Authors:** Shroque Zaher, Farah Otaki, Nabil Zary, Amina Al Marzouqi, Rajan Radhakrishnan

**Affiliations:** 1 College of Medicine, Mohammed Bin Rashid University of Medicine and Health Sciences, Dubai, United Arab Emirates; 2 Division of Pathology, Al Qassimi Hospital, Sharjah, United Arab Emirates; 3 Strategy and Institutional Excellence, Mohammed Bin Rashid University of Medicine and Health Sciences, Dubai, United Arab Emirates; 4 Institute for Excellence in Health Professions Education, Mohammed Bin Rashid University of Medicine and Health Sciences, Dubai, United Arab Emirates; 5 College of Health Sciences, University of Sharjah, Sharjah, United Arab Emirates

**Keywords:** Interprofessional education, IPE, Professional identity, Teamwork, Situated learning theory, Middle East and North Africa, United Arab Emirates, Value-based health care

## Abstract

**Background:**

The value of interprofessional education (IPE) in nurturing healthcare professionals, and in shaping their professional identities, and their attitudes towards interdisciplinary teamwork and collaboration is established in the literature. IPE is an emerging concept in the Middle East and North Africa (MENA) region and is new to the United Arab Emirates (UAE). To date, the applicability and feasibility of IPE and of the corresponding collaborative practice in MENA countries remain largely unexamined.

**Purpose:**

To investigate the effect of one of the first experiences of IPE in the UAE, which was purposefully designed in alignment with the principles of the Situated Learning Theory (SLT), on the readiness for interprofessional learning and collaboration among students of various healthcare disciplines in the UAE.

**Methods:**

A pre-post intervention quantitative research design was adopted for this study. The intervention focused on communication skills, and consisted of 2 tailor-made case-based scenarios. A total of 90 students (40 medical, 16 nursing, 16 pharmacy, and 18 physiotherapy), spread across two sessions (1 session per academic year across 2 academic years), took part in the IPE intervention. Readiness for Interprofessional Learning Scale (RIPLS) was used as the pre- and post- intervention assessments; aggregate data was analyzed using SPSS.

**Results:**

Of those who participated in the intervention (across both rounds), 77 participants responded to the pre-assessment (85. 6%) and 84 responded to the post-assessment (93. 3%). The IPE intervention under investigation significantly increased the level of readiness to engage in cross-disciplinary learning and collaboration among participating health professions’ students. In terms of the subscales, the participants’ openness to engage in teamwork was raised and their professional identity was fostered. Yet, no statistical significance around clarity of roles and responsibilities was detected.

**Conclusion:**

The findings of this study encourage other universities in the MENA region to adopt IPE to improve future health professionals’ capacity to develop shared understanding and mutual respect within cross-disciplinary teams. This, ultimately, feeds into improved quality of care and patient outcomes.

**Supplementary Information:**

The online version contains supplementary material available at 10.1186/s12909-022-03571-9.

## Introduction

Interprofessional education (IPE) is an inter-collaborative approach to develop healthcare students as future interprofessional team members, and is a practice promoted by the World Health Organization (WHO) as well as by various other international organizations such as the Canadian Interprofessional Health Collaborative (CIHC), the European Interprofessional Education Network, and the UK Centre for the Advancement of Interprofessional Education [1, 2].

Today’s patients have complex health needs and typically require more than a single discipline to address issues regarding their health status to attain improved health outcomes. Many of the patients’ adverse events and poor health outcomes may be attributed to misunderstandings and/ or poor communication among members of the interprofessional teams. Even though physicians and nurses work together, their academic courses are typically separate, and their training in effective strategies of communication and of care provision is often postponed to future professional practice [1, 3]. Studies support the introduction of interprofessional education at the start of healthcare students’ professional education rather than at the end [4, 5]. This is believed to prevent the formation of negative interprofessional attitudes [6].

Relational Coordination (RC) is a concept that appears to be central to IPE. It is defined as a mutually-reinforcing process of interaction between communication and relationships carried out for the purpose of task integration [7]. The RC theory suggests that for a team to be effectively coordinated, there is a need for the development of shared knowledge and understanding among its team members, as well as for the team’s relationships to be built on common goals and mutual respect [8]. This highlights the importance of communication and relating to one another for the purpose of task integration [9]. Relationships help shape the communication through which coordination occurs, and hence it is of utmost importance that emphasis is placed on teamwork and collaboration in the training of students in healthcare professions.

Effectively leveraging certain existent theories of education in designing and implementing IPE hold the potential of raising the reliability and robustness of the respective interventions [10, 11]. As such, Situated Learning Theory (SLT), which was initially proposed by Jean Lave and Etienne Wenger in the late 1980s, is suitable for application in this context. The concept of SLT relates to the conceptualization that learning occurs within authentic context, culture, and activity. It promotes the idea that students learn better in collaborative group settings and when the activities are based on real-life experiences. Knowledge needs to be presented in authentic contexts- settings and situations, that would typically require deploying that knowledge. Social interaction and collaboration are essential components of SLT; learners become involved in a “community-of-practice” which embodies certain beliefs and behaviors to be acquired. As the beginners or novices move from the periphery of a community to its center, they become more active and engaged within the culture and eventually assume the role of experts [12].

It is established that IPE has the potential to positively influence attitudes and perceptions towards interprofessional collaboration, and to increase the effectiveness of clinical decision-making and ultimately the quality of care [13, 14]. Many of the key competencies for IPE relate primarily to teamwork, including skills in setting shared goals, communication, knowledge of roles and responsibilities, and negotiation for conflict resolution [1, 9, 15].

Understanding one’s own role and that of other professionals in the healthcare team is critical in IPE. As students become more immersed in their own education, they are likely to gain a better and more comprehensive understanding of their role in the interdisciplinary healthcare team. Establishing a strong IPE foundation and opportunities for interprofessional learning within healthcare education is therefore paramount, as has long been emphasized by the WHO [2, 16]. Yet, many health professionals have had little or even no exposure to IPE activities during their own training, and many clinical sites in which faculty oversee training lack robust or explicit examples of interprofessional team-based care [17].

Interprofessional collaboration is an emerging concept in the MENA region, and is new to the United Arab Emirates (UAE) [18–22]. Literature from this region has highlighted context-specific barriers to implementation of IPE. Among the greatest challenges in the region to IPE implementation, is the gender segregation due to cultural barriers, and the limited dedicated time to establish IPE activities for faculty members. There is also the variation in efforts directed towards IPE, across differing healthcare professionals, and the logistic difficulties in planning, implementing, and evaluating IPE [18, 23]. Other challenges that are reported include the lack of objective clinical performance assessment methods, and difficulties in tracing the impact of IPE in terms of outcomes of care. In addition, it was also previously suggested that differences among some of the professions with respect to professional identities, and roles and responsibilities, could be potential hurdles to the realization of IPE [19, 24]. In the Kingdom of Saudi Arabia (KSA), the lack of IPE experts was identified as a significant challenge. Apparently, most faculty members lack the skills to teach IPE-focused curriculums. The inflexibility in the content of the medical curricula is believed to also contribute to the difficulties of offering IPE experiences, where healthcare profession students do not have sufficient time to participate in IPE-based learning opportunities [20]. For pharmacists in the MENA region in particular, the contextual barriers previously reported upon include the existence of a hierarchical structure, and the lagging societal image and marginalized contribution of pharmacists. Other challenges reported upon, in relation to pharmacists, encompass weak sense of professional identity, resistance within healthcare teams to develop the role and responsibilities of pharmacists, and the heterogenous background of healthcare professionals [25]. Most of the literature from the region is focused on evaluating the readiness of students for IPE learning. Only a few institutions have implemented structured IPE programs which encompass several healthcare disciplines. As for the UAE, formal structured experiences of IPE are deficient, and literature pertaining to IPE, specific to the UAE, is lacking.

In this paper, we describe one of the first structured experiences of IPE in the UAE, conducted at the Mohammed Bin Rashid University of Medicine and Health Sciences (MBRU) in collaboration with the University of Sharjah (UoS). This intervention was based on the principles of the SLT with the understanding that “real” learning can only happen when it is contextual (i.e., simulating an authentic experience that will engage the learners in complex, realistic, and problem-centered activities while recasting the role of the teacher to a facilitator) [26–28]. As such, the IPE intervention, described in this paper, builds upon previously generated knowledge and recommendations around the subject matter [20]. First, this intervention constitutes an example of how IPE is incorporated into health professionals’ curricula with the intention of graduating students ready for collaborative practice in the workforce. Second, the respective IPE intervention’s learning objectives are based on shared competency domains. Third, developing and implementing this IPE intervention was based on a reliable theoretical framework, namely: SLT.

Given the value of IPE in nurturing healthcare professionals, in shaping their professional identities and attitudes toward interdisciplinary teamwork and collaboration, and ultimately in improving patient outcomes of care [29], the purpose of this research study was to assesses the effect of an innovative IPE intervention, that was designed in alignment with SLT, on healthcare students’ self-assessed readiness (i.e., knowledge, skills, and attitudes) to collaboratively engage and learn with students of other healthcare disciplines. Accordingly, this study’s research questions are as follows:

- How efficacious was this IPE intervention at raising the level of self-assessed readiness for interprofessional learning and collaboration among the cross-disciplinary student participants?
- How did this IPE intervention affect the students’ perception of teamwork and collaboration, professional identity, and roles and responsibilities in the context of interprofessional learning and collaboration?

## Methods

### Context of the study

The study was carried out at MBRU in collaboration with the UoS. MBRU is one of 8 medical colleges in the UAE and one of two in Dubai. MBRU was established in 2016, and currently offers a 6-year Bachelor of Medicine, Bachelor of Surgery (MBBS) program [10, 30, 31] as well as postgraduate degrees in dentistry [11, 32, 33], nursing, and biomedical sciences. The first 3 years of the respective MBBS program are preclinical and the remaining years are spent in the clinical setting. Being a young university, MBRU is focused on innovation and research, not least so in the educational setting. In line with this, one of the longitudinal themes of the MBBS program is enhancing multidisciplinary teamwork in students, which constituted the basis of this study’s intervention. At the time of this study, 77% of the student body were female and 33% were UAE nationals. They were of 26 nationalities.

As for the UoS, it was established in 1997 and offers 110 programs across multiple academic disciplines. It offers both undergraduate and postgraduate degrees in medicine, nursing, pharmacy, and other allied health sciences such as physiotherapy. Both universities share a vision of graduating future doctors with the necessary interpersonal skills that would enable comprehensive multidisciplinary patient care.

### IPE Intervention

As part of the Neurosciences Course which takes place in the second semester of the third year of the MBBS program of MBRU, all enrolled third year medical students were required to attend an IPE session. This session was designed, implemented, and monitored and evaluated in alignment with the preset MBBS program outcome of promoting collaborative teamwork amongst students. Besides the MBRU MBBS participants, students from the UoS were recruited, where students, from each of the following disciplines: nursing, physiotherapy, and pharmacy, were invited to participate in the learning intervention. The students were recruited and selected from two differing universities because the respective MBBS is the only undergraduate program offered at MBRU. To ensure that the level of learning (i.e., undergraduate or postgraduate) does not play a confounding role in the relationship between the disciplines and the perceived level of readiness for IPE, other undergraduate-level programs were sought. To do so, the research investigators visited the UoS and met with the relevant discipline leads. Email communication was then sent out to all the enrolled students and those who expressed an interest were recruited (i.e., convenience sampling). A total of 90 students (40 medical, 16 nursing, 16 pharmacy, and 18 physiotherapy), spread across two sessions (1 session per academic year across 2 academic years), took part in the IPE intervention. In each of the 2 sessions, the students were split into eight groups. To each group of MBRU MBBS students, student representation was added from the schools of pharmacy, nursing, and physiotherapy from the UoS. The session was facilitated by two faculty members: a professor of pharmacology (RR) and an assistant professor of pathology (SZ). Prior to the session, students were asked to complete the Readiness for Interprofessional Learning Scale (RIPLS) questionnaire, and again, immediately after the session. In turn, the RIPLS data, collected from the two rounds of the intervention, was aggregated.

The session comprised of an initial introduction to the learning concepts of inter-professional healthcare teams and collaborative patient-centered care. The preset learning outcomes were designed based on Miller’s pyramid of clinical competence [34]. This theory proposes that learners move from a knowledge phase (knows and knows how), through a demonstration phase (shows how), and finally to a competent phase of ‘doing’. We believe IPE to be a continuum of competence that requires students to progress through these phases to adequately provide care in interprofessional settings. As such, the following three core competency domains of the Interprofessional Education Collaborative (IPEC) were used (https://www.ipecollaborative.org/ipec-core-competencies#:~:text=The%20IPEC%20panel%20identified%20four,teamwork%20and%20team%2Dbased%20care):

- Competency 1: Work with individuals of other professions to maintain a climate of mutual respect and shared values (i.e., values/ ethics for interprofessional practice)
- Competency 2: Use the knowledge of one’s own role and those of other professions to appropriately assess and address the healthcare needs of patients, and to promote and advance the health of populations (i.e., roles/ responsibilities)

Competency 3: Communicate with patients, families, communities, and professionals in health and other fields in a responsive and responsible manner that supports a team approach to the promotion and maintenance of health and the prevention and treatment of disease (i.e., interprofessional communication) Accordingly, the objectives of the session were as follows:

- Define interprofessional collaborative practice
- Discuss the benefits of interprofessional collaborative practice including the impact on quality and safety of patient care
- Describe key elements of effective interprofessional team-based care
- Discuss factors that may influence interprofessional collaboration
- Describe the roles, responsibilities, and abilities of various healthcare professions involved in collaborative work, including their training and respective scopes of practice
- Describe one’s own professional role in relation to collaborating with other healthcare professionals

Basing the development and delivery of the IPE intervention on the SLT theoretical framework enabled for coordinated design and implementation in terms of objectives, content, complexity, and delivery. In line with the principles of SLT [27], the session was based on realistic case-based problems with students split into teams, and the session facilitated by instructors, ensuring that their role was not that of a ‘teacher’ but that of a ‘coach’. Emphasis was placed on fostering meaningful interactions between the students within a cross-disciplinary team setting. As the session was part of the neuroscience course, the theme of the case studies were pain and stroke, respectively. Collins (1988) defined situated learning most simply as: ‘*the notion of learning knowledge and skills in contexts that reflect the way the knowledge will be useful in real life’.* As *such, t*he case studies which were constructed by both facilitators (SZ and RR), consisted of a short case history of a patient including history of presenting complaint, past medical history, drug history, and social history. This was followed by a series of questions for the students to discuss in groups. These questions were intentionally designed to generate discussions that are of relevance across all disciplines (Additional files 1 and 2). These questions included:

1. What is the immediate management of this patient?
2. What are the long-term sequelae of this event?
3. How can this patient be managed in the long-term and which professions will play a role?
4. How can the effect of another similar event be minimized for this patient?
5. What other health and social concerns are of importance to this patient?
6. What is the long-term management plan of this patient?

It was emphasized to the students that the focus was on engagement in a clinical discussion to appreciate perspectives from different disciplines and how this can add to their own body of knowledge. Determining the correct factual clinical material was secondary to the interprofessional discussion. As such, students were able to connect their prior knowledge with authentic and contextual learning, and were challenged to use their critical thinking in a cooperative setting.

### Research design

A pre-post intervention quantitative research design, with a single arm [35], was adopted for this study to assess the effectiveness of the IPE intervention in raising the level of self-assessed readiness for interprofessional work among the student participants. The strength of this design lies in the captured temporality, which enables examination of how a particular outcome is impacted by the intervention. Therefore, perceptions of the same group of students were measured both before and after the intervention. This study design is commonly adopted in health professions’ education research [36]. The study was conducted in two rounds over 2 years (each for a different group of participants). Students filled-out the RIPLS questionnaire before and after the sessions. Ethical approval for the study was granted by the MBRU, Institutional Review Board (Reference # MBRU-IRB-2020–016).

### Data collection

The data was collected using the RIPLS questionnaire. The version used was the Readiness for Interprofessional Learning Survey (RIPLS) questionnaire (Latrobe Community Health Service, 2009) which was adopted from the original RIPLS survey [37]. The students were given hardcopies of the survey right before they started the session and again right after they were done with the learning and development opportunity. An informed consent form was appended to the respective surveys. Participation was completely voluntary; the participants had complete autonomy to decide whether, or not, to participate in the respective study. Participants had the right to withdraw at any point in time and to refrain from answering any question(s). Data was anonymous; no personal identifiers were collected. There were neither penalties for non-participation nor incentives for participation.

RIPLS [38, 39] is a self-reporting tool that assesses perceptions of healthcare students' knowledge, skills, and attitudes regarding readiness to learn with other healthcare professionals. It is an internationally-recognised survey tool, which has been validated for use in the postgraduate context [40]. It is composed of 19 components that are measured using a 5-point Likert-type scale. The 19 components are divided into three validated subscales. The first one is the “Teamwork/ Collaboration” subscale which assesses the extent to which the participant values cooperative learning and respecting students from other healthcare professionals. The second one is the “Professional Identity” subscale which measures the tendency of the participant to value and benefit from collaborative relationships with other healthcare professionals. As for the third one, it is the “Roles and Responsibilities” subscale, and measures the practical application of interprofessional skills with other healthcare professional students.

Two out of those three subscales are further divided into two segments. The Teamwork/ Collaboration subscale has the “Need for positive relationships between professor and other healthcare students” and “Acquisition and effectiveness of teamworking skills” segments. As for the Professional Identity subscale, it has “Positive” and “Negative” segments. Scores can be generated for the segments, subscales, and overall rating. Higher scores indicate more readiness to engage in interprofessional learning experiences. It is worth noting that given the inverse nature of the Negative Professional Identity segment of the tool, the Likert-type scale of its components (10 through 12) were coded as such: 1: Strongly Agree, 2: Agree, 3: Neutral, 4: Disagree, and 5: Strongly Disagree.

### Data analysis

The quantitative data was descriptively analyzed using IBM SPSS Statistics Version 27. For each of the demographic variables, the number of cases, and frequencies and valid percentages were calculated. For each of the 19 quantitative components of the tool, the mean and standard deviation were calculated. An overall score of readiness was calculated (1 through 19), along with scores for each of the 3 subscales of the tool: Teamwork and collaboration (1 through 9), Professional identity (10 through 16), and Roles and responsibilities (17 through 19). Additional scores were calculated for the two segments of the Teamwork and collaboration subscale: Acquisition and effectiveness of teamworking skills (1 through 6), and Need for positive relationships between professionals and other healthcare students (7 through 9), and for the two segments of the Professional identity subscale: Negative (10 through 12) and Positive (13 through 16).

The validity tests of Cronbach’s Alpha and the Principal Component Analysis (PCA) were performed to check the adapted tool’s internal consistency and external variance.

For the inferential analyses, to select the appropriate tests, a test of normality was conducted for the data of each of the 19 components, and for all eight scores (overall, 3 subscales, and 2 segments within each of 2 of the subscales). The data of each of the 19 components, independently, and all the scores turned out to be not normally distributed. Accordingly, Mann–Whitney tests were used to compare the scores, and each component independently, pre- and post- intervention.

The scores, and each component independently, were compared across the demographic variables, as well: Age, Gender, and Discipline. For the dichotomous variable: Gender, Mann–Whitney test was used. As for the other two demographic variables, Kruskal- Wallis tests were conducted.

## Results

Forty-five students participated in each round of the IPE intervention. Of the total 90 participants, 77 completed the pre-intervention questionnaire and 84 post-intervention. As such, the overall response rate was 89.4% (Table 1).

**Table 1:** Response rates pre- and post- intervention in both rounds

Round	Pre			Post			Total		
	Number of Responses	Number of Participants	Response Rate	Number of Responses	Number of Participants	Response Rate	Number of Responses	Number of Participants	Response Rate
1st	43	45	95.6	45	45	100	88	90	97.8
2nd	34	45	75.6	39	45	86.7	73	90	81.1
Total	77	90	85.6	84	90	93.3	161	180	89.4

As illustrated in Table 2, the Age of the responders, who reported on this variable, ranged between 19 and 25, with the biggest portion of responders in the 20 years age group (31.1% in the pre-assessment and 35.1% in the post-assessment). Participants were predominantly female (74.7% in the pre-assessment and 71.1% in the post-assessment). In terms of Discipline, most of the responders were in Medicine (44.2% in the pre-assessment and 47.6% in the post-assessment), followed by Pharmacy, Physiotherapy, and Nursing, consecutively.

**Table 2:** Output of descriptive analysis of demographics data

Variable	Values	Pre		Post	
		**Frequency**	**Percent**	**Frequency**	**Percent**
**Age**	**25**	2	2.7	2	2.7
	**24**	5	6.8	1	1.4
	**23**	8	10.8	8	10.8
	**22**	6	8.1	8	10.8
	**21**	19	25.7	18	24.3
	**20**	23	31.1	26	35.1
	**19**	11	14.9	11	14.9
	**Total**	**74**	**100.0**	**74**	**100.0**
**Gender**	**Female**	56	74.7	54	71.1
	**Male**	19	25.3	22	28.9
	**Total**	**75**	**100.0**	**76**	**100.0**
**Discipline**	**Medicine**	34	44.2	40	47.6
	**Nursing**	13	16.9	13	15.5
	**Pharmacy**	15	19.5	16	19.0
	**Physiotherapy**	15	19.5	15	17.9
	**Total**	**77**	**100.0**	**84**	**100.0**
**Completed questionnaire before**	**Yes**	2	2.6	16	20.0
	**No**	75	97.4	64	80.0
	**Total**	**77**	**100.0**	**80**	**100.0**
**Time since completion of questionnaire**	**1–3 Month(s)**	1	50.0	6	60.0
	**3–6 Months**	1	50.0	2	20.0
	**1–2 Year(s)**	0	0	1	10.0
	**2–3 Years**	0	0	1	10.0
	**Total**	**2**	**100.0**	**10**	**100.0**
**Experienced interprofessional teaching before**	**Yes**	9	11.8	10	13.9
	**No**	67	88.2	62	86.1
	**Total**	**76**	**100.0**	**72**	**100.0**

A total of 75 responders in the pre-assessment and of 64 responders in the post-assessment indicated that this was the first time they complete a RIPLS questionnaire. In the pre-assessment, 1 responder reported completing it 1–3 Month(s) ago and 1 responder reported completing it 3–6 Months ago. As for the post-assessment, 6 responders reported completing it in 1–3 Month(s), 2 in 3–6 Months, and 1 in each of the remaining categories: 1–2 Year(s) and 2–3 Years. For 88.2% of the responders to the pre-assessment and 86.1% of those to the post-assessment, this intervention constituted their first exposure to interprofessional teaching.

The reliability score of Cronbach’s Alpha for the adapted RIPLS questionnaire was 72.4%. The percentage of the overall score was 86.9% pre-intervention and 89.9% post-intervention, as per Table 3. According to the PCA, 87.4% of the variance can be explained by the instrument (*P* < 0.001) which means the instrument is not only reliable but also valid to measure what it is intended to measure.

**Table 3:** Output of descriptive quantitative analysis of all the components and scores, pre- and post-intervention

Subscale	Segment	Component	Pre			Post		
			**Mean (± SD)**	**Percentage of the Mean**	**Category**	**Mean (± SD)**	**Percentage of the Mean**	**Category**
**Teamwork and Collaboration**	**Teamworking Skills**	1.Learning with other students will help me become a more effective member of a healthcare team	4.8 (0.5)	95.6	SA	4.8 (0.5)	95.8	SA
		2.Patients would ultimately benefit if healthcare students worked together to solve patient problems	4.8 (0.4)	95.8	SA	4.9 (0.3)	97.6	SA
		3.Shared learning with other healthcare students will increase my ability to understand clinical problems	4.7 (0.5)	94.6	SA	4.8 (0.5)	95.2	SA
		4.Learning with healthcare students before qualification would improve relationships after qualification	4.7 (0.6)	94	SA	4.8 (0.5)	95.8	SA
		5.Communication skills should be learned with other health care students	4.8 (0.5)	96.6	SA	4.8 (0.5)	96.6	SA
		6.Shared learning will help me to think positively about other professionals	4.6 (0.6)	92	A-SA	4.7 (0.6)	94.2	SA
		**Segment 1A Score**^b^	28.4 (2.2)	94.77	SA	28.8 (2.6)	95.9	SA
	**Positive Relationships**	7.For small group learning to work, students need to trust and respect each other	4.7 (0.6)	94.6	SA	4.8 (0.4)	96.4	SA
		8.Team-working skills are essential for all health care students to learn	4.6 (0.6)	92.4	A-SA	4.8 (0.4)	95.8	SA
		9.Shared learning will help me to understand my own limitations	4.9 (0.3)	97.6	SA	4.8 (0.6)	95.8	SA
		**Segment 1B Score**	14.2 (1.1)	94.9	SA	14.4 (1.2)	95.9	SA
	**Subscale 1 Score**^b^		42.7 (3.1)	94.8	SA	43.2 (3.6)	95.9	SA
**Professional identity**	**Negative**	10.I do not want to waste my time learning with other healthcare students^a,b^	4.2 (0.9)	84.2	A-SA	4.6 (0.8)	91	A-SA
		11.It is not necessary for undergraduate healthcare students to learn together^a,b^	4.0 (1.1)	80.6	A	4.5 (0.9)	89.2	A-SA
		12.Clinical problem-solving skills can only be learned with students from my own department^a^	3.6 (1.4)	72	A	3.7 (1.4)	73.8	A
		**Segment 2A Score**^b^	11.8 (2.7)	78.9	A	12.7 (2.3)	84.7	A-SA
	**Positive**	13.Shared learning with other healthcare students will help me to communicate better with patients and other professionals	4.5 (0.7)	90.6	A-SA	4.7 (0.7)	93.6	A-SA
		14.I would welcome the opportunity to work on small-group projects with other healthcare students^b^	4.4 (0.9)	88	A-SA	4.8 (0.5)	95.2	SA
		15.Shared learning will help to clarify the nature of patient problems^b^	4.3 (0.9)	86.8	A-SA	4.8 (0.5)	95.2	SA
		16.Shared learning before qualification will help me become a better team worker^b^	4.5 (0.7)	90	A-SA	4.7 (0.6)	94.2	SA
		**Segment 2B Score**^b^	17.6 (2.9)	88	A-SA	18.9 (2.1)	94.3	SA
	**Subscale 2 Score**^b^		29.4 (4.2)	84.1	A-SA	31.6 (3.6)	90.2	A-SA
**Roles and Responsibilities**		17.The function of nurses and therapists is mainly to provide support for doctors^b^	4.6 (0.7)	92.8	A-SA	4.8 (0.5)	96.2	SA
		18.I am not sure what my professional role will be	2.3 (1.2)	45.8	U	2.1 (1.3)	42.6	D-U
		19.I have to acquire much more knowledge and skills than other healthcare students	3.5 (1.1)	70.2	A	3.8 (1.1)	76.6	A
		**Subscale 3 Score**	10.4 (1.9)	69.5	A	10.7 (2)	71.3	A
***Overall Score***^b^			82.5 (6.5)	86.9	A-SA	85.4 (7.2)	89.9	A-SA

*D* Disagree, *U* Undecided, *A* Agree, *SA* Strongly Agree ^b^Statistically significant difference pre-post assessment: the post assessment turned-out to be significantly higher than the pre assessment

^a^Values corrected to match the rest of the scale

^b^Category flipped to match the rest of the scale

The post-assessment overall readiness score, with a mean of agreement of 85.4(± 7.2), was significantly higher than its pre-assessment counterpart, with a mean of agreement of 82.5(± 6.5) (*p* = 0.001).

The Teamwork and collaboration subscale score was significantly higher in the post-assessment, relative to the pre-assessment (*p* = 0.031). The Teamworking skills segment score was also significantly higher in the post-assessment (*p* = 0.037), while there was no significant difference in the Positive relationships segment score. In terms of the components of the Teamwork and collaboration subscale (1 through 9, independently), none showed significant difference between the pre- and post-assessments.

As for the Professional identity subscale score, with a mean of agreement of 31. 6(± 3.6), it was significantly higher than the same score assessed pre-intervention, with a mean of satisfaction of 29.4(± 4.2) (*p* = 0.001). The two segments of this score: Negative and Positive professional identity, were also significantly higher in the post-assessment relative to the pre-assessment (*p* < 0.05). In terms of the components of the Professional identity subscale (10 through 16, independently), components 12 and 13 assessed post-intervention were not significantly different than their pre-intervention assessment. As for the remaining components (10 and 11, and 14, 15, and 16), they significantly increased in the assessment after the intervention (*p* < 0.01).

As for the Roles and responsibilities subscale, the analysis showed no statistically significant difference between pre- and post-assessment; among the components of the respective subscale (17 through 19, independently), only 17 significantly increased in the assessment after the intervention (*p* = 0.034).

In the pre-assessment, the medicine participants scored significantly higher than those of the rest of the disciplines in the Teamworking skills segment. The same category of participants also scored higher, relative to others, in relation to the following components: 5 and 6, and 13 (*p* < 0.05). With regards to component 2, pharmacy participants scored significantly higher compared to the participants of the other disciplines (*p* = 0.049). There was no statistically significant difference between the scores of female and male participants, and across the various Age groups. In terms of components, there was significant difference across the scores of the Age groups for components 1, 11, 16, 18, and 19 (*p* < 0.05). For components 1 and 11, 25 years age group was significantly higher than the rest of the Age groups; 24 years age group higher for 16, 19 years age group for 18, and 21 years age group for 19.

In the post-assessment, the nursing participants scored significantly higher than those of the rest of the disciplines in the Roles and responsibilities subscale, only (*p* < 0.05). Moreover, there was no statistically significant difference between the scores of female and male participants, and across the various Age groups.

## Discussion

The current study revealed that the IPE intervention under investigation significantly increased the level of readiness to engage in cross-disciplinary learning and collaborations among the participating health professions’ students. This intervention constituted a novel experience for most of the participating students. Its efficaciousness, in the context of the study, namely: UAE, encourages other universities in the region (i.e., MENA) to adapt such interventions to increase students’ level of readiness to engage in cross-disciplinary learning and collaborations. This will need to be preceded with efforts directed towards building the IPE capacity among faculty, who deliver the medical and health sciences curriculums in the MENA region. The importance of IPE needs to be highlighted and the competences needed to deliver such content need to be proactively developed [3]. For that purpose, regional medical education leaders can learn from similar faculty development experiences in other contexts. A widespread initiative in the United States, in 2012–2013, included an extensive faculty development course to prepare faculty for IPE. This experience generated an evidence-based guide which briefly describes the faculty development program and identifies key lessons learned from the initiative. They highlighted the importance of peer-learning, adapting the curriculum to fit local context, experiential learning, and ongoing mentoring, especially in relation to actual participation in IPE activities [41]. It is recommended that faculty development initiatives aim to bring about change at the individual and the organizational levels [42]. Accordingly, it would be worthwhile to concurrently target diverse stakeholders, and several content areas, including but not necessarily limited to: interprofessional education and collaborative patient-centred practice, teaching and learning, and leadership and organizational change. It is better for those faculty development opportunities to take place in diverse settings, using a variety of educational strategies.

Moreover, the evaluation tool utilized in this study, namely: RIPLS [39], proved to be internally reliable and externally valid in evaluating the students’ responsiveness to IPE in the context of the current study. This finding is crucial, given the previously identified limitations of this tool, where a study from Norway demonstrated that RIPLS has substantial “ceiling effects”. This term refers to a measurement limitation that occurs when the highest possible score on a measurement instrument is reached, decreasing the likelihood that the instrument has accurately measured the intended domain [43]. Moreover, Schmitz et al. [44] highlighted that the constructs measured in IPE by the RIPLS are inter-linked and therefore not distinct which make high inter-correlations among these items, nearly inevitable. Other studies identified similar limitations from a psychometrics’ perspective, where problems at item-level and subscale results of the RIPLS were observed [45].

There is increasing interest in the theoretical underpinning of IPE. Several key theories have been used in IPE curriculum design, many of which take a constructivist approach. SLT, which was the basis of the current study’s intervention, is one such example. Constructivism emphasizes how social encounters influence learners’ understanding and development [46]. SLT emphasizes that learning occurs in the context of the experience, and places great emphasis on relationships and interactions with others in building understanding and developing the role of the individual within the greater community [47]. Relying on SLT enabled identifying and in turn circumventing context-specific barriers. It became obvious to the two facilitators that the implicit hierarchical structure among healthcare professions which is present internationally is actually more prominent in the MENA region. The relative lag of societal image of allied healthcare professionals relative to physicians and the marginalization of their contribution to the delivery of care surfaced as influencing factors, as well. This sheds light on a byproduct of this intervention where engaging allied healthcare professionals in IPE actually lifted-up their morale and contributed to breaking down the silos of the different disciplines. This effect was further fostered by the intentional socializing (i.e., icebreaking activity) where the participants got to know each other, on a personal level, prior to the actual intervention.

RC argues that for a team to be effectively coordinated, there is a need for shared knowledge and understanding among its members, as well as for all the entailed relationships to be built upon shared goals and mutual respect [7]. It is established in the literature that communication and other interpersonal skills among healthcare professionals are essential for the quality of healthcare delivery [48]. Strong relationships in teams are expected to contribute to effective service delivery and improved patient health outcomes [49]. In the setting of the current study, students of differing healthcare disciplines were able to collaborate and exchange knowledge. This enhanced their teamwork and fostered their professional identities, which is expected to translate, on the long run, in better outcomes of care, which is particularly relevant to value-based health care systems [50]. It is worth highlighting, over here, the variation in openness to IPE among the differing disciplines (in the MENA region) that the two facilitators observed. When lobbying for the intervention in the various universities of medicine and health sciences, nursing educators expressed excitement. As for those who teach medical students, they seemed reluctant to the subject matter. This could be attributed to the hierarchical structure, where the allied health professionals who are perceived (especially in the MENA region) to be lower in rank are logically affected more. Interestingly, nursing students appear to significantly benefit from such IPE interventions. Relative to that of the rest of the disciplines, the level of readiness for inter-disciplinary teamwork among nursing students, in relation to the roles and responsibilities, was the highest after the intervention under investigation in the current study. Along those lines, a previously conducted study revealed a significant improvement in RIPLS scores with nursing students following an interdisciplinary learning intervention but not for any of the other disciplines evaluated [51]. In the current study, it was clear to the facilitators that medical students were addressing the cases from a purely technical perspective. As for the rest of the participating students, besides the medical technicality, they were able to factor into their perception other aspects of the patient care. Their contributions in the discussion appeared as eye-openers to the medical students, and as such helped in widening the scope of the learning experience.

It is established that readiness for interprofessional learning is fundamental to healthcare team development [52]. Hence, identifying factors that influence the students’ readiness for interprofessional learning is fundamental to developing learning strategies targeted to improve teamwork and quality of care, and ultimately patient health outcomes [51]. An evaluation of attitudes towards IPE, among pharmacy students in the MENA region, showed that most respondents perceive IPE to be important [21]. Another similar study looking at the perception of students training in medicine, nursing, and laboratory sciences in the KSA showed similar findings. It revealed that most respondents consider themselves to be ready for engaging in multidisciplinary collaborations [53]. The facilitators in the current study became cognizant from their firsthand experience of the importance of factoring into the medical and health sciences curriculums dedicated time to IPE; this requires flexibility which is particularly relevant to the MENA region since the whole IPE field is still lagging.

The receptiveness to IPE tends to vary across disciplines and from one context to another. In this study, prior to the intervention, participants training in medicine scored higher in terms of readiness for cross-disciplinary work. This might be attributed to the longitudinal themes that are integral to most of the contemporary medical programs (including the MBBS program that the medical students participating in this study are enrolled in), where competencies such as professional identity and practice (which includes but is not limited to interprofessional practice) are systemically nurtured throughout the respective programs. Another study showed that pharmacy and dietetics students demonstrated a higher level of readiness for interdisciplinary learning compared to other disciplines [51]. In a study conducted among South Korean health profession students, the nursing students’ perception of the importance, preference, and effectiveness of IPE was the highest, whereas medical students’ perception was the lowest. All students (i.e., medical, nursing, and pharmacy) perceived their present level to be lower than that required for each interprofessional competency [54].

As previously outlined, specific subscales of the RIPLS include teamwork and collaboration, professional identity, and roles and responsibility. Comparison of the subscale domains by discipline is useful in understanding views and attitudes that may be characteristic of a specific discipline and may provide an explanation for the differences observed between the disciplines for RIPLS composite score. The intervention under investigation significantly raised the level of readiness regarding teamwork and collaboration. It also increased the readiness in relation to acquisition and effectiveness of teamworking skills. These findings are in alignment with the work of Lairamore et al. (2013) who reported improved RIPLS scores following a case-based interprofessional intervention across all disciplines [5].

It is well established that teams offer the promise to improve clinical care because they can aggregate, modify, combine, and apply a greater amount and variety of knowledge to make decisions, solve problems, generate ideas, and execute tasks more effectively and efficiently than any individual working alone [55]. Accordingly, introducing students to interdisciplinary collaboration early-on in their education trajectory will help foster the concept of a team mentality and comprehensive patient care, where each medical professional holds a piece to the puzzle. As such, the IPE intervention investigated in this study was intentionally carried-out in the medical students’ pre- clinical years. Non-medical students often find it difficult to achieve equal interaction as they expect medical students to lead the conversation. This narrative is particularly prevalent in the MENA region and presents a greater incentive for breaking down the silos of different disciplines [24, 53]. It will also be important, since IPE is a novel concept in the MENA region, to introduce professional development programs to raise awareness, among faculty members, about IPE, along with preparing them to teach it. Moreover, ensuring the IPE intervention’s incorporation into the respective curricula (with the necessary assessment methods), as per the experience in the current study, is important. As such, the students will have reserved time to acquire the corresponding competences.

Interventions such as the one investigated in this study holds the potential to significantly foster the participants’ professional identity. This appeared to be the case through decreasing their negativity around the subject matter and improving the extent of positivity associated with their identity due to such cross-disciplinary interventions. Interestingly, although the intervention improved the state of readiness among the participants in relation to the first two subscales: teamwork and collaboration, and professional identity, as mentioned above, there was no statistical significance around roles and responsibilities. The only exceptional component of this scale was: “The function of nurses and therapists is mainly to provide support for doctors”. Along those lines, another study aimed at evaluating the medical students’ readiness and perception of IPE in a medical college in the KSA revealed a similar pattern and recommended for shared academic events to focus on clarifying the roles and responsibilities of medical students in multi-disciplinary healthcare teams [53].

The current study is characterized by a few limitations, which can be perceived as opportunities for further research. To start with, although the intervention under investigation was mandatory for the medical students, the recruitment of participants from the other programs relied on convenience sampling. This might have skewed the results since most probably the students who enrolled in the intervention were the ones who are motivated to learn. For future studies, we recommend for the intervention to be integral to the various curriculums, where all the students, and not solely the medical ones, will be mandated to take part of the intervention. This investigation was deductive in nature and relied solely on quantitative data. It would be useful for future studies to be exploratory in nature, where focus group sessions can be conducted to better understand why there are differences across healthcare disciplines in terms of receptiveness to IPE. Moreover, the study results are based on a single intervention that was conducted in two rounds for different batches. Although they were meant to be congruent and (as previously mentioned) all the intricacies of the intervention were kept constant, slight variations were inevitable given the elapsed time between both rounds (i.e., one academic year). In addition, to maintain the participants’ anonymity, there were no personal identifiers recorded in the pre- and post- assessments of this study. This has disabled linking the data, on the participant-level, which is why this study’s analysis was performed on an aggregate level. It is recommended for future studies to be based on a longitudinal design where the pre- and post- assessment are linked perhaps through assigning unique identifying numbers to the participants (which will maintain their anonymity). Such a design can be further stretched to trace the long-term impact of IPE initiatives on quality of care and in turn patient health outcomes. For the participants of the current study, the researchers intend to conduct a follow-up investigation to assess how this IPE intervention affected their engagement in cross-disciplinary teamwork and collaborations in their clinical years and internships, and as they progress in their career paths. It is worth investing time and resources to develop context-specific clinical performance assessment methods. Also, the data collection was not electronic and relied on hardcopies of the respective surveys. Consequently, not all participants responded to all questions. Also, although the version of the RIPLS used included an open-ended question towards the end, the fact that it was not electronic seemed to affect the likelihood that the students actually provide narrative feedback, where only a handful of students wrote anything in the respective section. As such, there was no mentioning of any qualitative component in the current study.

## Conclusion

IPE interventions, designed in alignment with SLT such as the one investigated in the current study, hold the potential to significantly increase the participating health profession students’ level of readiness to engage in cross-disciplinary learning and collaborations. It is recommended for other universities in the MENA region to adapt IPE to improve future health professionals’ capacity to develop shared understanding and mutual respect within cross-disciplinary teams, which ultimately feeds into improved quality of care and patient outcomes. This will require investing resources in building the capacity of faculty members around interprofessional learning and collaboration among healthcare professionals. Moreover, the medicine and health sciences students need to have dedicated time for IPE, preferably integrated into the curriculum. Finally, efforts need to be directed on institutional, national, and regional levels to flatten the implicit hierarchical structure among healthcare professionals.

## Supplementary Information

**Additional file 1.**

**Additional file 2.**

## Data Availability

All data generated or analysed during this study are included in this published article and its supplementary information files.
