# Supplementary material for: Effect of introducing interprofessional education concepts on students of various healthcare disciplines: a pre-post study in the United Arab Emirates
Source: BMC Med Educ. 2022 Jul 2;22:517. doi: 10.1186/s12909-022-03571-9 (PMC9250223; doi:10.1186/s12909-022-03571-9)
Supplement: Supplementary file 1 — Additional file 1. [file 12909_2022_3571_MOESM1_ESM.docx]

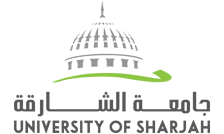

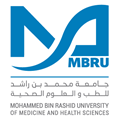


**MEDC 3636: Neurosciences Course**

**Interprofessional Team-based Learning Session**

**Date: May 1, 2019**

**Venue: Lecture Hall 5, MBRU**

**Coordinators: Dr. S Z and Prof. RR**

**Case 2: Pain Patient**

***Chief Complaint (CC):***

Abdulla is a 50-year-old- male working in a farm in Fujairah. His GP referred him to a neurologist in Dubai for chronic back pain radiating down his right leg that is affecting his work now.

***History of Present Illness (HPI):***

Abdulla is having back pain that started radiating to his right leg for the last 5 years. He is a farm worker and is required to lift heavy objects as part of his job. He also has pain in his left hip region that does not radiate, but gets worse on days when he has lift heavy objects. He has been treated by a local GP for the last 5 years with no much improvement to his complaints, and the pain has started affecting his work. When he visited his home country during last summer, one of his relatives who is a physician advised him to see a neurologist. Thus he was referred to a neurologist by his GP. He has been told by his GP that he should not take ‘strong’ medications for pain as they can cause addiction. The patient is generally scared of side effects and would like to know if his conditions could be treated non-pharmacologically.

***Past Medical History (PMH)***

He has a history of hypertension, type II DM, hypercholesterolemia and atrial fibrillation.

***Current Medications:***

Nifedipine 10 mg PO, TID

Atorvastatin 20 mg PO, QD

Metformin 500 mg PO, QD

Paracetamol PO, PRN

Ibuprofen PO, PRN

Vitamin B Complex 1 tab PO, QD

St John’s wort 2 capsules QD

***Allergies:***

Allergic to ACEIs. Had swelling of the tongue last time when he started captopril and his GP took him off it.

***Family/Social History (FH/SH):***

Abdulla is a married expatriate working in Fujairah, and his immediate family lives in Bangladesh. He has two grown-up children who are in college and he is the sole bread-winner for the family. He smokes 2-3 packets of cigarette in a week, but does not drink. He doesn’t do any regular exercise as he thinks that he doesn’t need it because his daily work involves a lot of physical exertion. He occasionally takes over-the-counter herbal medications for general well-being and depression that are mostly suggested by his friends.

***Physical Examination:***

50-year-old south-east Asian

VS: BP-140/90. Pulse-83. Temp-97.2. Ht-5’6’’, Wt-140 lb

**Labs:**

Glucose-109 mg/dL (Fasting)

Total Cholesterol-150 mg/dL

Triglycerides-77 mg/dL

HDL- 41 mg/dL

LDL- 100 mg/dL

Na – 140 mEq/L

K – 6 mEq/L

***Major Discussion Points***

1. Assessment of the patient
2. Plan for short-term and long-term management
3. Recommendations for the patient
4. Non-pharmacological management of pain
5. Social and financial implications of his condition
