# Supplementary material for: Effect of introducing interprofessional education concepts on students of various healthcare disciplines: a pre-post study in the United Arab Emirates
Source: BMC Med Educ. 2022 Jul 2;22:517. doi: 10.1186/s12909-022-03571-9 (PMC9250223; doi:10.1186/s12909-022-03571-9)
Supplement: Supplementary file 2 — Additional file 2. [file 12909_2022_3571_MOESM2_ESM.docx]

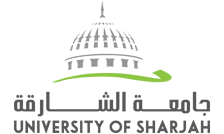

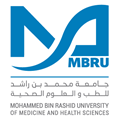


**MEDC 3636: Neurosciences Course**

**Interprofessional Team-based Learning Session**

**Date: May 1, 2019**

**Venue: Lecture Hall 5, MBRU**

**Coordinators: Dr. S.Z and Prof. R.R**

**Case 1: Stroke Patient**

***Presenting complaint (PC):***

Acute onset left face droop, left arm and left weakness

***History of presenting complaint (HPC):***

63-year-old male named Ahmed presents to the emergency room with acute onset left face droop, left arm, and leg weakness. The sudden onset of symptoms occurred at the post office where he works part time. One of his co-workers called 911. On the way to the hospital, the ambulance team evaluate Ahmed’s neurological deficits. The ambulance team notify the receiving hospital.

Upon Ahmed’s arrival to the hospital, the ER physician proceeds to gather the patient’s medical history from his daughter Fatima who accompanied him in the ambulance.

***Past medical history (PMH)***

Uncontrolled hypertension (non-compliant with antihypertensive medications).

Recent diagnosis of Type II diabetes

Hypercholesterolemia

***Drug history (DH)***

Aspirin 80 mg QD

Atorvastatin 20 mg PO, QD

Captopril 20 mg PO, BD

Metformin 500 mg PO, BD

No known allergies

***Family history (FH)***

Both of Ahmed’s parents passed away from myocardial infarctions when they were in their late 60s.

***Social history (SH)***

Ahmed is a smoker, usually smoking about a pack and half each day. He had tried to quit several times, he had trouble abstaining for longer than a week. He leads a sedentary lifestyle that has contributed to his excess weight. He dislikes physical activity and his idea of “exercise” is watching sports on television. His wife has recently died and he is living alone. His only family is his daughter Fatima, who is working full time.

***Physical examination***

Male, Arab of Palestinian origin

63 years old

Weight: 115kg

Height: 167cm

**Vital signs:**

BP-152/95. Pulse-77. Temp-97.4.

**Lab results**

Glucose-112 mg/dL (Fasting)

Total Cholesterol-185 mg/dL

Triglycerides-77 mg/dL

HDL- 40 mg/dL

LDL- 129 mg/dL

Na – 139 mEq/L

K – 4.5 mEq/L

***Major Discussion Points***

1. What is the immediate management plan for this patient?
2. What are the long-term sequelae of this event?
3. How can this be managed in the long-term and which professions will play a role?
4. How can the chances of another similar event be minimised in this patient?
5. What other health and social concerns are of importance in this patient?
